# Therapeutic Role of Zinc Supplementation in Children Hospitalized with Pneumonia

**DOI:** 10.7759/cureus.4475

**Published:** 2019-04-16

**Authors:** Ghulam Shabbir Laghari, Zahid Hussain, Muhammad Taimur, Nasir Jamil

**Affiliations:** 1 Pediatrics, Liaquat University of Medical and Health Sciences, Jamshoro, PAK; 2 Pediatrics, National Institute of Child Health, Karachi, PAK; 3 Internal Medicine, Dow University of Health Sciences, Karachi, PAK; 4 Miscellaneous, University of Karachi, Karachi, PAK

**Keywords:** zinc, pneumonia, acute respiratory illness, pakistan, paediatrics

## Abstract

Introduction

Pneumonia is one of the major causes of death in children younger than age five, especially in developing countries. The World Health Organization recommends children from a developing country take zinc supplements. We conducted this study to explore the efficacy of zinc supplementation in alleviating symptoms and shortening of hospital stay in children with pneumonia.

Materials and methods

We conducted this prospective, randomized controlled trial in the Department of Pediatrics, Civil Hospital, Jamshoro. We included 100 children of both genders randomized into two equal groups of zinc-supplemented and non-zinc-supplemented study groups after informed consent was obtained from the parents and legal guardians. The participants were aged 28 days to five years and admitted in the hospital with pneumonia. We monitored for hypoxia, tachypnea, chest indrawing, and cyanosis, and we recorded the total length of hospital stay for each group.

Results

We found no significant difference in symptom changes (i.e., hypoxia, tachypnea, chest indrawing, and cyanosis) between the zinc and non-zinc groups. However, hospital length of stay was significantly shorter for patients in the zinc-supplemented group compared to the non-zinc-supplemented group.

Conclusion

Zinc supplementation did not yield a statistically significant reduction in symptoms in children with severe pneumonia. Zinc supplements given during an acute episode are not beneficial in short-term clinical recovery from severe pneumonia in hospitalized children.

## Introduction

Acute respiratory infections (ARIs) are the main cause of morbidity worldwide, and among ARIs, pneumonia is one of the major causes of death in children younger than age five, especially in developing countries. The increased risk for ARIs in developing countries can be linked to poverty, lower education levels, low birth weight, malnutrition, and lack of breastfeeding [1]. According to a 2013 National Demographics and Health Survey of Pakistan, 16% of children under age five exhibited symptoms of ARI in the two weeks preceding the survey. These symptoms were more common in children younger than age two as compared to those aged from two to five years (20% vs. 13%, respectively). Furthermore, pneumonia was determined to be the cause of death in around 91,000 children [2]. The World Health Organization and the United Nations Children’s Fund recommend that children in developing countries should intake zinc supplements for 10 to 12 days as follows: 10 mg daily for infants younger than six months and 20 mg daily for infants older than six months to hasten recovery from severe pneumonia in developing countries. This can be linked to boosted immunity in response to zinc supplementation [3]. Zinc strengthens the immune system via its role in the maintenance of epithelial and tissue structure by promoting cell growth and reducing apoptosis. It also has antioxidant properties which protect against free radical damage sustained during inflammatory responses [4].

Various studies have highlighted the role of zinc in the management of severe pediatric pneumonia. Brooks et al. suggested that zinc supplemented in children younger than age two suffering from severe pneumonia resulted in a significant reduction in the severity of symptoms including tachypnea, anorexia, and restlessness; it also shortened the duration of hospital stay [5]. An Indian study showed that, compared to standard treatment, adding zinc to the treatment regime in children admitted with severe pneumonia resulted in faster improvement of symptoms and shortened duration of hospitalization [6]. However, there is still not enough local data to establish the efficacy of zinc in alleviating pneumonia symptoms in Pakistan.

The present study was conducted to determine the efficacy of zinc supplementation in alleviating symptoms and shortening of hospital stay in pediatric pneumonia.

## Materials and methods

We conducted a prospective, randomized controlled trial conducted in the Department of Pediatrics, Civil Hospital, Jamshoro. The study included children of both genders, aged 28 days to five years who were admitted to the hospital with pneumonia after informed consent from the parents and legal guardians.

A physical examination was performed to assess nasal flaring, respiratory rate per minute, breathing effort for the presence of chest indrawing, and peripheral cyanosis. The chest was auscultated for crepitations. Oxygen saturation was measured via pulse oximeter (hypoxia was taken as oxygen saturation <92%). Children with respiratory rate higher than the normal range for their age [7], with or without cough, and crepitations on chest auscultation were diagnosed with pneumonia, and children with a minimum of one danger sign (i.e., chest indrawing, cyanosis, grunting, and an inability to feed) were categorized as “severe pneumonia.” Children with no crepitations or any other finding on chest auscultation were excluded. Children whose parents/guardians did not consent to the trial after brief detailing were also excluded. A total of 100 children were randomized into two groups using a random number generator: one group receiving 20 mg/day oral zinc supplements, the other receiving no zinc supplements or placebo. Both groups were managed with standard pneumonia protocol including antimicrobial therapy, respiratory support, and fluid resuscitation.

Gender, age in months, and previous hospital admission due to respiratory illness in the previous month were recorded. The respiratory rate, arterial oxygen saturation levels, and signs of respiratory distress (i.e., nasal flaring, chest indrawing, cyanosis, inability to feed, and tachypnea) were noted at the time of admission and 48 hours of intervention in both groups. The duration of the hospital stay was noted for every child. Data were entered and analyzed using IBM Statistical Package for the Social Sciences (SPSS) Statistics for Windows, version 21.0. (IBM Corp., Armonk, NY, US). Mean and standard deviation were calculated for continuous variables including age and respiratory rate. Categorized variables were presented as frequencies and percentages. The comparison was done within both groups at admission and 48 hours after admission. Dependent T-test was applied to compare means, and chi-square was applied to compare percentages. P-values of ≤0.05 were considered statistically significant.

## Results

There were 50 children in each group. The zinc group had 60% (n = 30) male children, and the non-zinc group had 66% (n = 33) male children. The mean age of the entire sample was 29 ± 7 months. Age, gender, and previous hospital admission due to respiratory illness in the last month for both study groups are shown in Table 1.

**Table 1:** Patient demographic characteristics at the time of admission SD: Standard deviation.

Patient characteristics	Zinc group	Non-zinc group
Gender		
Male	30 (60%)	33 (66%)
Female	20 (40%)	17 (34%)
Age in months (mean ± SD)	27 ± 6	30 ± 4
Previous hospital admission due to respiratory illness in the last one month		
No episode	27 (54%)	21 (42%)
1-2 episodes	18 (36%)	11 (22%)
>2 episodes	5 (10%)	18 (36%)

For both study groups, respiratory rate, oxygen saturation, and clinical signs of respiratory distress were recorded at the time of admission and at 48 hours of intervention. The mean respiratory rate, frequency of hypoxia, and frequency of signs of respiratory distress significantly improved after 48 hours of intervention in both the zinc group and non-zinc group as shown in Table 2. The mean duration of hospital stay was significantly shorter in the zinc-supplemented group than the non-zinc group (Table 3).

**Table 2:** Change in patient characteristics from admission to 48 hours in zinc and non-zinc group SD: Standard deviation; NA: Not applicable.

Patient characteristics	Zinc group (n = 50)			Non-zinc group (n = 50)		
	At admission	At 48 hours	p-value	At admission	At 48 hours	p-value
Respiratory rate (mean ± SD)	49.69 ± 6.27	39.87 ± 4.72	<0.0001	51.05 ± 4.47	45.28 ± 2.58	<0.0001
Hypoxia (arterial O_2 _saturation <92%)	16 (32%)	3 (6%)	0.001	18 (36%)	10 (20%)	0.07
Signs of respiratory distress						
Nasal flaring	22 (44%)	5 (10%)	0.0001	25 (50%)	9 (18%)	0.0007
Chest indrawing	21 (42%)	7 (14%)	0.0001	23 (46%)	10 (20%)	0.005
Cyanosis	2 (4%)	NA	NA	2 (4%)	NA	NA
Inability to feed	8 (16%)	NA	NA	10 (20%)	2 (4%)	0.01
Tachypnea	39 (78%)	13 (26%)	<0.00001	42 (84%)	19 (38%)	<0.00001

**Table 3:** Mean duration of hospital stay in zinc and non-zinc group SD: Standard deviation.

	Mean ± SD duration of hospital stay in hours	p-value
Zinc group	3.12 ± 0.99	0.01
Non-zinc group	3.57 ± 0.81	

## Discussion

Both study groups demonstrated improvements in respiratory rate, oxygen saturation, and respiratory distress after 48 hours of intervention. The zinc-supplemented group had a shorter mean duration of hospital stay compared to the non-zinc-supplemented group. Our findings were similar to those of a meta-analysis of seven randomized controlled trials in which the therapeutic role of zinc with standard therapy was compared with standard therapy alone in 1,066 children from developing countries admitted for acute lower respiratory tract infections. There was no significant difference in the resolution of severe illness measured using symptoms such as hypoxia, chest indrawing, and tachypnea [8].

Das et al. found no significant difference between zinc and non-zinc groups in terms of hospital stay, while in our study, hospital stay was significantly shorter in the zinc group compared to the non-zinc group [8]. Even when compared to neighboring countries such as Bangladesh, India, and Nepal, a systemic review of four articles in 2011 with 3,200 children failed to prove a significant effect on the clinical recovery of pneumonia when zinc is added to the treatment regime [9]. In 2016, another meta-analysis of nine studies failed to established zinc as an adjunct therapy to antibiotics in children suffering from pneumonia. There was no significant reduction in time to recovery, hospital length of stay, treatment failure, and change of antibiotic when compared to placebo [10]. Therefore, our study, compared with other meta-analyses and systemic reviews, suggests there is no therapeutic role of zinc in children hospitalized with pneumonia.

However, Brooks et al. [5] and Coles et al. [6] reported a significant improvement in symptoms in zinc-supplemented participants as compared to placebo. A review of six randomized double-blind and placebo-controlled trials of adjunct zinc therapy in patients with severe pneumonia failed to show significantly improved clinical outcomes; however, a significant reduction in mortality due to severe pneumonia was reported for the zinc-supplemented groups [11]. In another study, the zinc-supplemented group experienced reduced time for symptom resolution and had shorter hospital stay durations compared to patients receiving a placebo [12].

The evidence regarding the role of zinc as an adjunct therapy in children with severe pneumonia is controversial. As discussed above, where some major clinical trials have cast zinc in a promising light, others have shown no clinical benefit to patients. While this study reported robust results, it was not without certain limitations. The age of participants in this study was very diverse (from age 28 days to five years), and different ages have various presentations, severities, and outcomes for pneumonia. Younger children have more severe pneumonia than older children. Also, this study included clinical parameters but not serological characteristics of the children. This study did not report patient outcomes in terms of mortality and discharge. Future clinical studies should be designed to evaluate the role of zinc in severe pneumonia with the addition of assessing clinical, radiological, and serological parameters, and measure disease outcome in terms of the resolution of symptoms, duration of hospital stay, and mortality.

## Conclusions

Zinc supplementation provided no statistically significant reduction in symptoms of severe pneumonia in children under age five. Zinc supplementation given during an acute episode does not help in the short-term clinical recovery from severe pneumonia in hospitalized children.
